# Failure of introduction of food allergens after negative oral food challenge tests in children

**DOI:** 10.1007/s00431-015-2504-x

**Published:** 2015-03-13

**Authors:** J. P. M. van der Valk, R. Gerth van Wijk, Y. Vergouwe, N. W. de Jong

**Affiliations:** 1 Department of Internal Medicine, Section of Allergology, Erasmus MC, Rotterdam, The Netherlands; 2 Center for Medical Decision Making, Department of Public Health, Erasmus MC, Rotterdam, The Netherlands

**Keywords:** Children, Failed introduction, Food allergy, Oral food challenge test

## Abstract

One of the purposes to perform an oral food challenge (FC) test is to avoid unnecessary elimination of food allergens. In case of a negative FC test result, the food can be introduced. It is, however, unknown if patients act according to the outcome of the test. This study evaluates the rate of introduction of peanut, hazelnut, cow’s milk or hen’s egg allergens after a negative FC test. We investigated the introduction rate of children (0–18 years) with a negative FC test visiting the Department of Allergology, Erasmus Medical Centre Rotterdam from 2008 till 2013 and the factors that influence the rate of introduction. Patients were asked to complete a comprehensive questionnaire about their FC test. In total, 157 (38 % girls, mean age during challenge 6.9 years) participated in the study. Of these FC tests, 104 (56 %) were followed by a successful introduction, 30 (16 %) by a partly introduction (traces or processed foods) and 52 (28 %) by a failed introduction. Peanut and hazelnut showed a statistically significant lower successful introduction rate. Age, gender, symptoms during FC test, dietary advice and time period to introduction significantly influenced the rate of introduction. One fourth of the children with failure of introducing foods experienced symptoms during the introduction.

*Conclusion*: More than one quarter of all children with a negative FC test result did not introduce the food. The FC test in its current form does not achieve its objective for this group of children.

**Table:** 

**What is Known:** • *When the outcome of a food challenge test is negative, the food should be introduced in the diet of the child.* • *Failure of this introduction has negative consequences for the health of the child.*
**What is New:** • *Failure of introduction of foods after a negative challenge test is reported in almost 25 % of the challenged children.* • *Failure of introduction after a negative challenge test is significant associated with gender, age, allergens, symptoms during OFC (according to the parents), advice, time start eating the food, and symptoms during introduction.*

**What is Known:**

• *When the outcome of a food challenge test is negative, the food should be introduced in the diet of the child.*

• *Failure of this introduction has negative consequences for the health of the child.*

**What is New:**

• *Failure of introduction of foods after a negative challenge test is reported in almost 25 % of the challenged children.*

• *Failure of introduction after a negative challenge test is significant associated with gender, age, allergens, symptoms during OFC (according to the parents), advice, time start eating the food, and symptoms during introduction.*

## Introduction

Food challenge (FC) tests play an important role in the diagnoses of food allergy. FC tests are performed to determine the presence of food allergy and to substantiate the advice to eliminate or to introduce the food. If a FC test is negative, food can be introduced into the diet. The aim of this study is to assess the rate of introduction of peanut, hazelnut, cow’s milk or hen’s egg (hereafter referred to as ‘milk’ and ‘eggs’, respectively) after a negative FC test. Factors that may influence this are also studied. Furthermore, symptoms during the introduction at home are evaluated. Between the beginning of January 2008 and the end of December 2012, children aged 0–18 years with a suspected peanut, hazelnut, cow’s milk or hen’s egg allergy and a negative FC test for one or more of these allergens were included in this study regardless of sensitization to the offending allergen.

## Materials and method

All food challenges performed in the period 2008 till 2013 on the Department of Allergology, Erasmus Medical Centre Rotterdam, were analysed, using the database from the department. All children with a negative food challenge were approached to participate in this retrospective cross-sectional study. Either the parents of the children (0–15 years old) or the children themselves (16–18 years old) were asked to complete a comprehensive written questionnaire.

### Questionnaires

The questionnaire contained a total of 16 questions. The first part (six questions) concerned the successful or failed introduction of the challenged foods and the time elapsed between the FC test and introduction. In case of a failed introduction, the children/parents were asked to explain the reason of failure. This section of the questionnaire also addresses any symptoms during the FC informing the patient’s perspective (three questions). The second part of the questionnaire contained four questions concerning the received advice, understanding and agreement regarding the FC test results. The last part of the questionnaire (three questions) focussed on symptoms that emerged at home during the introduction period.

### SPT

The skin prick test (SPT) was performed by application of the extract on the skin of the volar aspect of the forearm. The extract was pierced thought the skin barrier with a lancet. A dilution buffer was used as negative control, and histamine (1 %) was used as positive control. The area of the urticae was determined by a scanning programme by using a scanner device (Hewlett Packard 2400c, Houston, TX, USA) and software earlier developed by Erasmus Medical Centre (PAAMOST). This programme counts the surface of the area and calculates the HEP index. This calculation is done by dividing the area of the wheal of the allergen by the area of the mean of two positive controls. This method was used because of high accuracy and reproducibility. A HEP index >0.21 is considered as positive [2].

### Food challenges

Open food challenge (OFC) tests and double-blind placebo-controlled food challenge (DBPCFC) tests performed in the past were analysed. In the OFC test, the child received an unmasked food, the suspected allergen, in increasing dosage with time intervals of 30 min. The same time and dosage schedule was used in the DBPCFC test, but the allergen was now processed in a matrix and the child received on 1 day the placebo and the other day the verum. Blinding was guaranteed for the physician, the nurse and the patient. Blinding was broken 24 h after the challenge. The validated and standardized food challenge material used in the FC test (DBPCFC and OFC) was prepared according to the recipe developed by Berber-Vlieg et al. in 2008 [10]. The food challenge test consisted of a six-step doses regime. Upon completion of the challenge test, the child had consumed 1.75, 3.5, 14, 70, 130 and 350 mg protein equivalent, as is cumulatively one half a cup of cow’s milk, one third hen’s egg, three peanuts or four hazelnuts. The protocol for assessing the outcome of the DBPCFC as published by Vlieg-Boerstra et al. was used [11], and the recommendations as described by Niggemann et al. were followed according to interpretation of clinical symptoms during FC test [7]. The challenged recipes are shown per allergen in Table 1. For introduction of the food after a negative FC test, patients were guided and advised in an optimal way by a dietician or physician.

**Table 1:** Doses schedule OFC and DBPCFC tests

Dose	Cow’s milk (ml)	Hen’s egg (mg)	Peanut (mg)	Hazelnut (mg)	Protein equivalent (mg)
1	0.05	13	6	12	1.75
2	0.1	27	12	25	3.50
3	0.4	108	48	100	14
4	2.0	538	241	500	70
5	10.0	2690	480	860	130
6	50.0	13460	1206	2500	350
Cumulative	½ Cup	1/3 Egg	3 Peanuts	4 Hazelnuts	

### Definition of food introduction

To measure the reasons for failure of introduction, we divided the children into three categories: those with a successful introduction, those with a partly successful introduction and those with a failed introduction. Successful introduction was defined as starting as well as continuing eating of the food. Partly successful introduction was defined as when the child consumed only traces or processed products. Children with a failed introduction did not eat the food at all or tried once and never consumed it thereafter.

Verbal informed consent was obtained; medical ethical review was not needed according to Dutch law in case of this questionnaire.

### Statistical analysis

We reported the patient and the study characteristics in mean values, ranges and proportions. The association between patient characteristics and the introduction were analysed with ordinal regression analysis given the ordinal value of introduction (successful, partly, failed). Significance was determined at *p* < 0.05. All analyses were performed with SPSS software, 20th edition.

## Results

### Patients

**Fig. 1:**
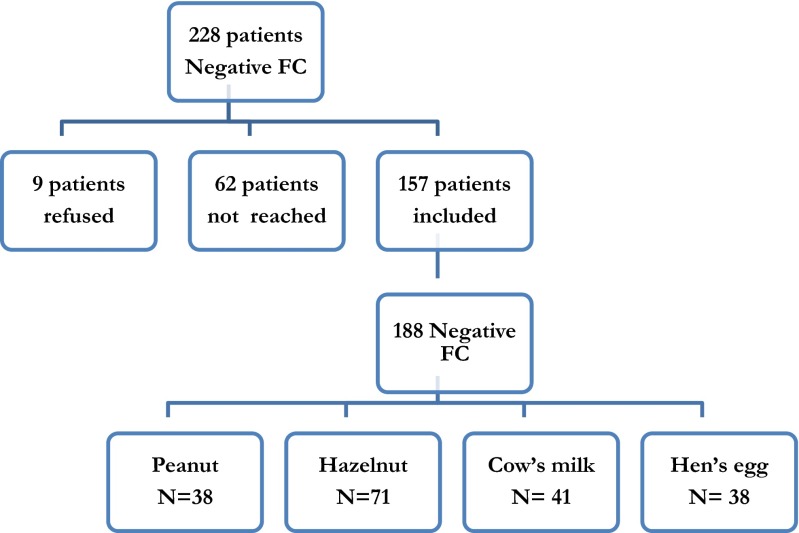
Results of inclusion

In this retrospective cross-sectional study, 188/269 FC tests with a negative outcome could be evaluated. In total, 343 FC tests were performed in the period 2008 till 2013 on the Department of Allergology, Erasmus Medical Centre. Of these 343 FC tests, 269 (78 %) had a negative outcome (egg 19 %, milk 22 %, hazelnut 41 % and peanut 20 %) (Fig. 1). These 188 FC tests were performed in 157 patients who were willing to participate in this study (62 % boys). One type of allergen was tested in 129 children, two different allergens were tested in 25 children, and 3 children had three FC tests with different allergens. The mean age during FC test was 6.9 years (range 0.6–17.1 years). Mean age of children who underwent milk and egg challenges was younger (4.6 and 6.4 years, respectively) than the children who were challenged with peanut and hazelnut (7.4 and 8.1 years, respectively). Most children were sensitized (sIgE or SPT) to the challenged food. The mean sIgE value of the sensitized patients was 8.53 IE/l (range 0.44–59) for milk, 11.57 IE/l (range 0.49–51.50) for egg, 14.99 IE/l (range 0.38–100) for peanut and 20.20 IE/l (range 0.36–100) for hazelnut. The mean SPT HEP index of sensitized patients was 1.18 (range 0.24–3.52) for milk, 1.12 (range 0.25–3.63) for egg, 1.56 for peanut (range 0.24–5.77) and 0.82 (0.25–2.75) for hazelnut. However, 16.6 % was not sensitized, and these children were tested because of a history of allergic or non-allergic symptoms after ingestion of the allergen in combination with fear to introduce the food. History of asthma was reported in the questionnaires in 40.1 %, hay fever in 47.1 %, and history of eczema in 86.6 % or another food allergy in 60.5 % of the children.

### Food challenge test

One hundred eight-eight negative food challenge tests could be analysed, 146 were DBPCFC tests and 42 were OFC tests. Five children had a repetition of the FC with the same allergen because of a failed introduction. The main reason for failure was an FC test performed too long ago, causing anxiety to introduce the food. The main reason to perform the peanut and hazelnut FC test was to establish the clinical relevance of sensitization. The milk and egg FC tests were mainly performed to establish whether a child had overgrown the allergy. The allergens tested were 38 (20.2 %) for peanut, 71 (37.8 %) for hazelnut, 41 for milk (21.8 %) and 38 (20.2 %) for egg.

### Questionnaires

In total, 186 of 188 questionnaires about the FC tests were completed by the 157 participating children (2 missing questionnaires). One hundred four (55.9 %) tests were followed by a successful introduction, 30 (16.1 %) by partly (traces or processed foods) introduction and 52 (28 %) by a failed introduction. The successful introduction rate for peanut and hazelnut was significantly lower in comparison with the successful introduction rate for milk and egg, even after correction for age (*p* = 0.001) (Table 2). In our study, 43 (40.2 %) FC tests with peanut and hazelnut were followed by a failed introduction in comparison with 9 (11.4 %) of the milk and egg FC.

**Table 2:** Potentially influencing factors of a failed introduction after a negative FC test

Potentially influencing factors	*N* (%)	*N* (%)	*N* (%)	
	Successful introduction	Partly* introduction	Failed introduction	Statistical significance (*p* value)
Total**	104 (55.9)	30 (16.1)	52 (28)	
Initial symptoms before FC test				0.309
Never eaten	45 (55.6)	10 (12.3)	26 (32.1)	
No	7 (46.7)	1 (6.6)	7 (46.7)	
Yes	49 (57.6)	19 (22.4)	17 (20.0)	
Gender				0.042
Girl	26 (44.1)	9 (15.2)	24 (40.7)	
Boy	57 (58.2)	17 (17.3)	24 (24.5)	
Age (year)				0.004***
0–4	44 (64.7)	9 (13.2)	15 (22.1)	
4–8	33 (54.1)	10 (16.4)	18 (29.5)	
≥9	27 (47.4)	11 (19.3)	19 (33.3)	
Allergens				0.001
Peanut	15 (39.5)	8 (21.0)	15 (39.5)	
Hazelnut	33 (47.8)	8 (11.6)	28 (40.6)	
Cow’s milk	28 (68.3)	9 (21.9)	4 (9.8)	
Hen’s egg	28 (73.7)	5 (13.1)	5 (13.2)	
Kind of FC test				0.596
DBPCFC	80 (55.6)	28 (19.4)	36 (25.0)	
Open	24 (57.1)	2 (4.8)	16 (38.1)	
Symptoms during FC according to the parents				0.005
No	92 (60.1)	25 (16.4)	36 (23.5)	
Yes	12 (36.4)	5 (15.1)	16 (48.5)	
Advice				0.054
Yes	59 (61.5)	17 (17.7)	20 (20.8)	
No	44 (50.0)	13 (14.8)	31 (35.2)	
Time to start eating the food				<0.001
Never started	0 (0.0)	0 (0.0)	27 (100)	
A week	84 (73.7)	14 (12.3)	16 (14.0)	
A month	14 (53.8)	6 (23.1)	6 (23.1)	
A year	3 (75.0)	1 (25.0)	0 (0.0)	
Forgotten by patient	3 (27.3)	5 (45.4)	3 (27.3)	
Symptoms during introduction				<0.001
Yes	15 (44.1)	7 (20.6)	12 (35.3)	
No	89 (71.2)	23 (18.4)	13 (10.4)	
Never started	0 (0.0)	0 (0.0)	27 (100.0)	

*Traces and processed food

**Two missing questionnaires (188–2 = 186)

***Based on continues variable

Some of the rows do not add up to 186 because of some missing data

**Fig. 2:**
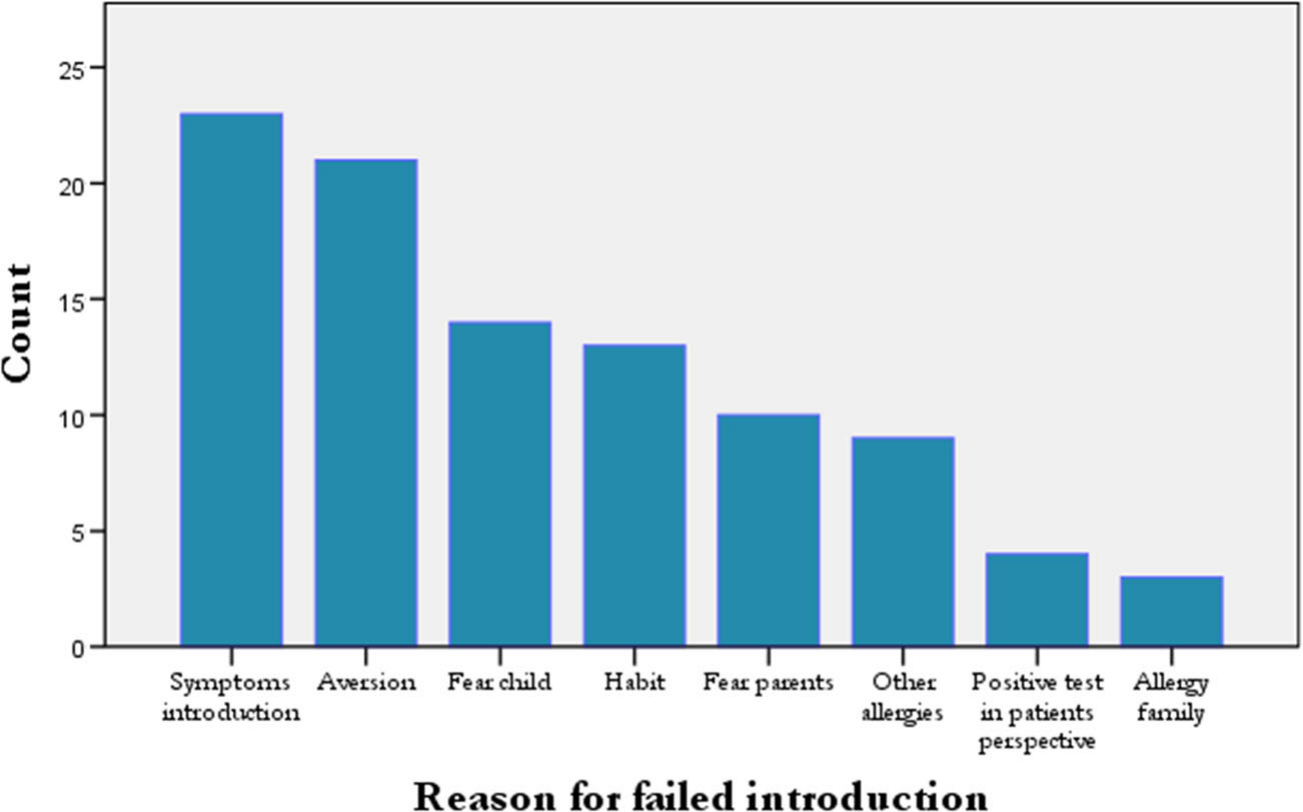
Main reason for a failed introduction according to the questionnaires

According to the questionnaires, the main reason for a failed introduction was a reaction occurring during the introduction at home (23 %), followed by aversion to the food (21 %), fear of the child (14 %), habit to avoid this food (13 %) and fear of the parents (10 %) (Fig. 2). Remarkably, 4 % of the parents were convinced that the FC test was positive, notwithstanding the clinician had issued the FC test as negative. More than 2 % of the children did not introduce the food within a year after the challenge, and 14.8 % never tried the tested food at home after a negative FC test.

### Statistical calculations

Boys showed a higher successful introduction rate than girls (*p* = 0.042). Successful introduction was associated with a lower age (*p* = 0.004). Initial symptoms before the FC test did not influence rate of introduction. Even initial symptoms related to anaphylaxis caused no higher failed introduction rate. There was no difference in success of introduction between OFC and DBPCFC. With regard to time between challenge and introduction of the food, complete introduction of the challenged food was most likely to succeed in children who had introduced the food fast after the FC test (within 1 week). The most successful introduction rate was achieved in children whose parents were advised to introduce the food (*p* = 0.004).

The rate of introduction was significantly lower if the child experienced symptoms during the FC test (*p* = 0.005). The rate of introduction was also significantly lower if the child experienced symptoms during introduction (*p* < 0.001). A quarter of the 28 % children with a failed introduction experienced reactions during the first ingestion and stopped the introduction consequently. The children experienced mainly skin reactions, followed by gastrointestinal and respiratory symptoms. Non-allergic reactions (headache and sore nose) were reported in two children. The majority of the reactions occurred at home and were related to the main component dose, followed by the processed dose or to pure allergens. Initial symptoms before the FC test had no effect on the introduction rate.

## Discussion

Our study shows a high failed introduction rate for milk, egg, hazelnut and peanut. Twenty-eight percent of the children failed to introduce the challenged food. Eigenmann et al. determined the proportion of failed introductions by questioning 73 patients after a negative FC test with different kinds of food. Failed introduction was reported in 25.4 % of the cases (18/71) [3], which is comparable with the test results of our study. The study of van Erp et al. determined the proportion of failed introduction in 103 children with a negative peanut challenge test. Introduction failed in 32 % of the children [9]. Flammarion et al. examined the consumption of the food after a negative FC test. They investigated the frequency of recurrent allergic reactions during introduction and its consequences on daily life for 67 children who underwent a total 110 FC tests. In this study, a successful introduction rate of 83 % has been reported [4]. Finally, Dambacher et al. have reported a successful introduction rate of 81 % (60 out of 74) for children with a cow’s milk allergy [1]. The high successful introduction rate of these latter two studies compared to our study might be due to the kind of allergen tested. The percentage of negative FC test with milk and egg are higher in the study of Flammarion et al. compared with our study. The study of Dambacher et al. included only negative FC tests with milk in contrast to our study with negative FC tests with milk, egg, peanut and hazelnut. To take the kind of allergen tested into account is important, because our study demonstrated that 43 (40.2 %) FC tests with peanut and hazelnut were followed by a failed introduction in comparison with 9 (11.4 %) of the milk and egg FC tests. Moreover, even after correction of age, the FC test for peanut showed significantly lower successful introduction than for milk and egg. The lower successful introduction for peanut and hazelnut could be caused by frequent publicity on the subject, which enhances awareness and fear for peanut allergy, as in daily life traces of peanut are difficult to avoid in the diet [3].

Indeed, successful introduction was associated with a significantly lower age. The successful introduction rate for boys was higher than that for girls. A significant (*p* = 0.026) higher rate of introduction for boys compared to girls was also reported by Eigenmann et al. [3]. We did not find an increase of the successful introduction rate after an OFC test in comparison with a DBPCFC test.

Symptoms during introduction at home had a significant influence on failed introduction, in one fourth of the children. This factor is also reported by van Erp et al., Eigenmann et al. and Flammarion et al. [3, 4, 9]. Adverse events during introduction after a negative FC test have been documented for failure in 12.7 % of the cases by Eigenmann et al. and in 5.5 % by Flammarion et al. A possible explanation for the relatively high occurrence of symptoms during introduction at home might be that the introduction dose was higher than the final dose of the FC test. All FC tests were performed with a recipe described in the study by Vlieg-Boerstra et al. [9]. The term failed introduction is thus debatable; the children who experienced symptoms during introduction might react to a higher eliciting dose than used in the challenge. Consequently, an open challenge with higher doses at the department should be performed to identify susceptibility to higher doses and at the same time convincing for the child and parent that the allergen is not harmful. Another reason for the more frequently occurring symptoms during introduction at home could be the differences in intrinsic and extrinsic factors between clinic and home and the food matrix [8].

Furthermore, the child and/or the parents of the child should be informed by the clinician at forehand about the indication for the FC test and the consequences of the outcome of the FC test. After the negative challenge, dietary advice is recommended, with explanation about reasons why and methods how, to introduce the food, as the introduction rate also depended on the given advice. A clear cut introduction scheme should be given to the parent or child, and a follow-up appointment to evaluate the success of introduction is mandatory. Most importantly, the parent and/or child need firstly to be convinced that the challenge results is negative, otherwise the chance that they will introduce the allergen in their diet is small. Taking fear away to ingest the food at home is important for the chance of a successful introduction. Failed introduction may lead to incomplete diets, missing essential nutrients and a lower quality of life [6].

Moreover, there is a chance of developing an acute allergic reaction after long time elimination [5]. The time period between challenge and introduction appeared also to be of great importance. This finding is not described in earlier studies and might be a useful recommendation for daily practice. We recommend to introduce the relevant food within 1 week after challenging the patient.

This is the largest study on the introduction rates of food after a negative FC test. As a result, we had the unique opportunity to investigate many influencing factors on the introduction rate. However, the retrospective design is a limitation of this study. Time span between food challenge and completion of the questionnaire is wide. It is obvious that children challenged in the end of 2012 filled out the questionnaire more correctly than children challenged in 2008. The multivariate model in the study of van Erp et al. showed that long interval between FC test and questionnaires was significantly associated with introduction failure in children with a suspected peanut allergy [9].

The questionnaire is not validated, and associations are mostly investigated with univariate analysis, because of the relative small groups.

In summary, we found a high rate of failed introduction after a negative food challenge. Fear is an important factor, most likely caused by assumed symptoms during the FC tests and symptoms during introduction of the food at home. For a successful introduction of food after a negative challenge, we recommend a higher total dose of the food in the challenge and a clear dietary advice with 1 week follow-up to guide introduction of challenged food.

## Abbreviations

DBPCFC: Double-blind placebo-controlled food challenge
FC: Food challenge
OFC: Open food challenge
